# Non-enzymatic reactions in biogenesis of fungal natural products

**DOI:** 10.1007/s11418-024-01797-z

**Published:** 2024-03-22

**Authors:** Shinji Kishimoto

**Affiliations:** Department of Pharmaceutical Sciences, University of Shizuoka, Shizuoka, 422-8526 Japan

**Keywords:** Cyclopenase, Diels–Alderase, Azlactone, Diastereomer, Spontaneous reaction

## Abstract

Fungi have long been regarded as abundant sources of natural products (NPs) exhibiting significant biological activities. Decades of studies on the biosynthesis of fungal NPs revealed that most of the biosynthetic steps are catalyzed by sophisticated enzymes encoded in biosynthetic gene clusters, whereas some reactions proceed without enzymes. These non-enzymatic reactions complicate biosynthetic analysis of NPs and play important roles in diversifying the structure of the products. Therefore, knowledge on the non-enzymatic reactions is important for elucidating the biosynthetic mechanism. This review focuses on non-enzymatic reactions we recently encountered during biosynthetic studies of four types of NPs (viridicatins, Sch210972, lentopeptins, and lentofuranine).

**Graphical abstract:**

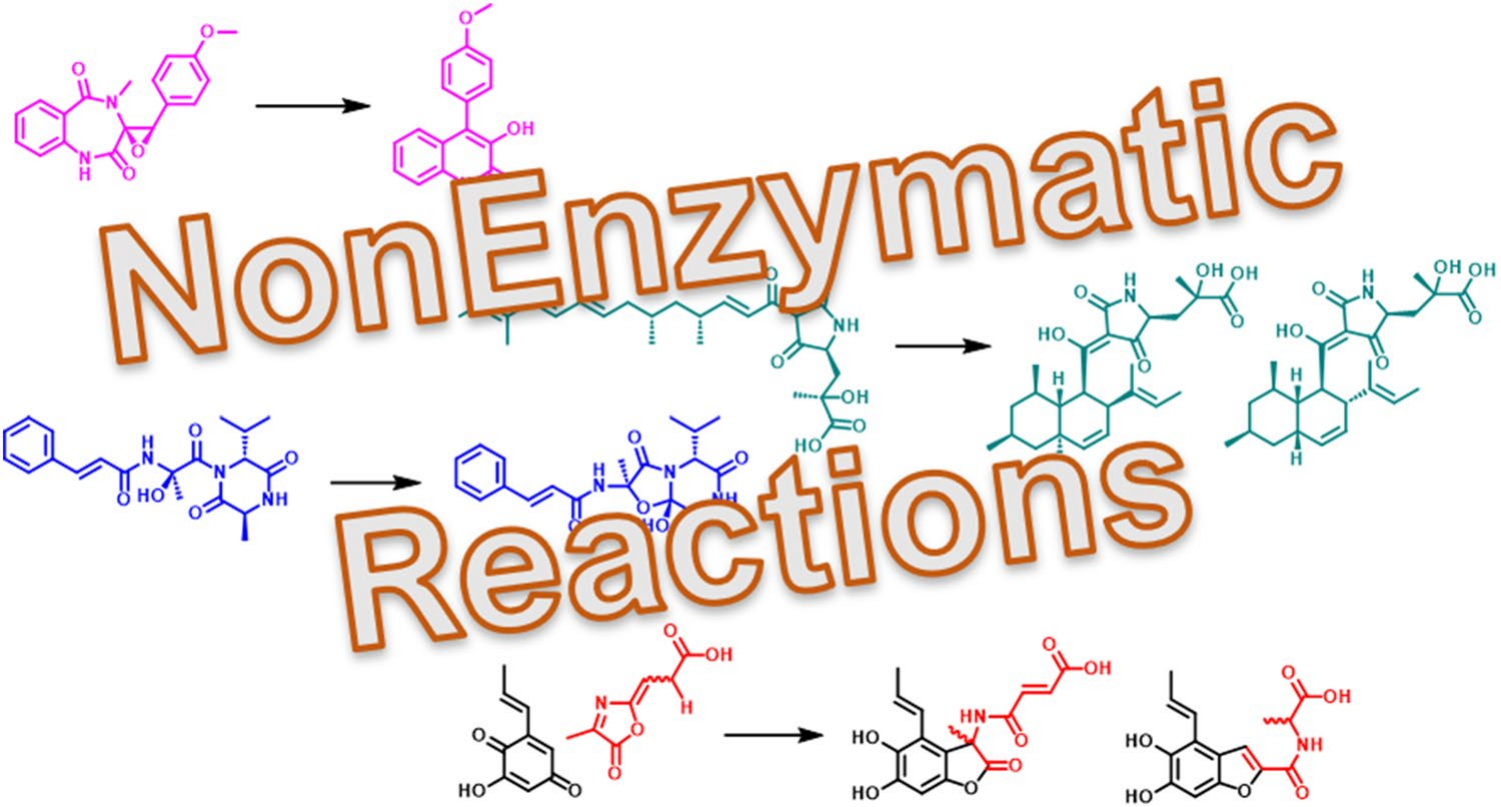

## Introduction

**Fig. 1:**
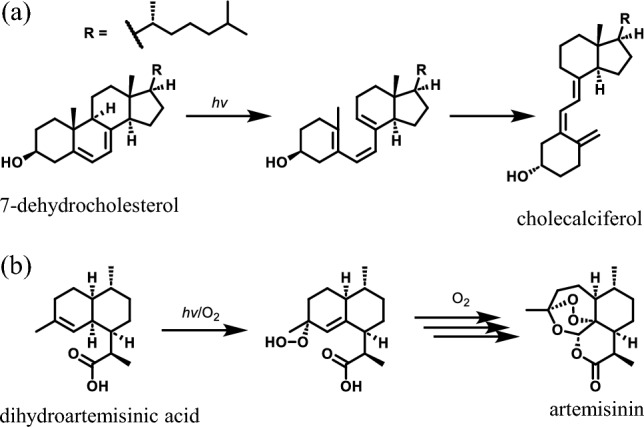
Spontaneous reactions involved in the formation of pharmaceutically important metabolites. **a** Photochemical production of cholecalciferol from 7-dehydrochoresterol. **b** Autoxidation of dihydroartemisinic acid to artemisinin

Fungi produce a wide range of natural products (NPs) such as polyketides [1], terpenes [2], non-ribosomal peptides [3] and hybrids of them [4, 5]. These compounds sometimes exhibit significant biological activities and thus fungi have been considered as attractive sources of pesticides and pharmaceuticals. Understanding the biosynthesis of NPs allows for the discovery of novel NPs through genome mining and the creation of modified NPs via genome engineering. As a result, the biosynthetic pathways of numerous important metabolites have been extensively researched and established so far [1–5]. Biosynthesis of NPs is usually catalyzed by sophisticated biosynthetic enzymes but not all of the reactions require enzymes. These chemical reactions proceeding without enzymes are called non-enzymatic reactions [6, 7]. One of nature’s most famous non-enzymatic reactions is photochemical conversion of 7-dehydrocholesterol to cholecalciferol, also known as vitamin D3 (Fig. 1a) [8]. Another example can be found in the formation of artemisinin from dihydroartemisinic acid although the mechanism remains unclear (Fig. 1b) [9]. These examples show that non-enzymatic reactions are not always undesired nor unnecessary for the producer and human beings. On the other hand, non-enzymatic conversion of NPs sometimes complicates biosynthetic analysis. In this review, recent discoveries of non-enzymatic reactions in the fungal biogenesis of NPs and related enzymatic reactions are introduced and discussed.

## Biosynthesis of viridicatins

**Fig. 2:**
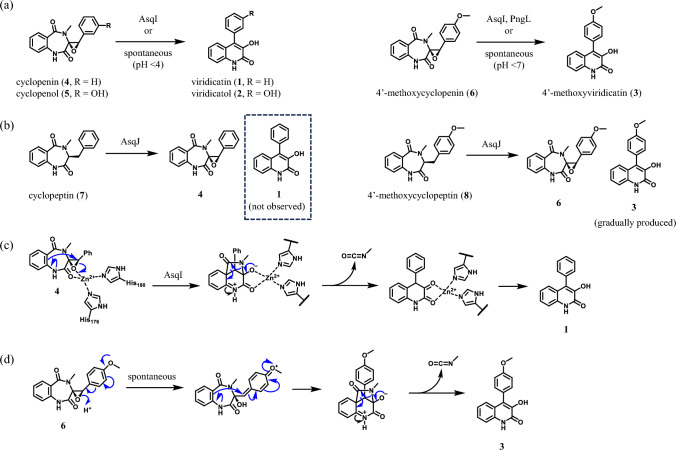
**a** Conditions for converting cyclopenins **4**–**6** to viridicatins **1**–**3**. **b** Production of **4** and **6** by AsqJ from cyclopeptin (**7**) and 4′-methoxycyclopeptin (**8**), respectively. **c** Mechanism of AsqI-catalyzed conversion of **4** to **1**. **d** Mechanism of spontaneous transformation from **6** to **3**

Viridicatin (**1**), viridicatol (**2**), and 4′-methoxyviridicatin (**3**) are fungal NPs produced by *Aspergillus* and *Penicillium* species [10–12]. The first report on the biosynthesis of these compounds was provided in 1967. Luckner showed that homogenized mycelia of *Penicillium viridicatum* could convert cyclopenin (**4**) and cyclopenol (**5**) into **1** and **2**, respectively [13]. The active material in the mycelia was deduced to be an enzyme and was named “cyclopenase”, which was not identified for a long time. In 2014, Ishikawa and co-workers reported that non-heme iron dioxygenase AsqJ could produce **4** and 4′-methoxycyclopenin (**6**) from cyclopeptin (**7**) and 4′-methoxy cyclopeptin (**8**), respectively (Fig. 2b) [14]. In this study, spontaneous formation of **3** from **6** was observed during AsqJ reaction. To the contrary, **1** was not produced from **4** in neutral pH conditions, indicating that cyclopenase is necessary for the conversion of **4** to **1** in nature. This observation encouraged us to search for the cyclopenase gene, and we found hemocyanin-like enzyme AsqI is encoded in the gene located near *asqJ*. Heterologously expressed AsqI converted **4** and **6** to **1** and **3**, respectively, thus AsqI was proved to be the missing cyclopenase (Fig. 2a) [15]. Crystallographic and biochemical analysis of AsqI revealed that a Zn(II) ion bound in the metal binding domain is responsible for the enzymatic reaction. Zn(II) ion acts as a Lewis acid to promote the ring opening of epoxide and induces the elimination of methylisocyanate from **4** to produce **1** (Fig. 2c). The reason why **6** spontaneously transforms to **3** but **4** does not transform to **1** without cyclopenase can be explained by the effect of the methoxy group of **6**. Delocalization of a lone pair of electrons on the methoxy group leads to the spontaneous opening of the epoxide ring (Fig. 2d) [14]. In the case of **4** and **5**, activation of epoxide with a strong acid (Lewis acid or Brønsted acid) can induce nonenzymatic transformation at room temperature. Bräuer and co-workers reported that **1** was also produced during the AsqJ reaction, which seems to be an accident caused by trichloroacetic acid they added after the reaction [16].

## Biosynthesis of Sch210972

**Fig. 3:**
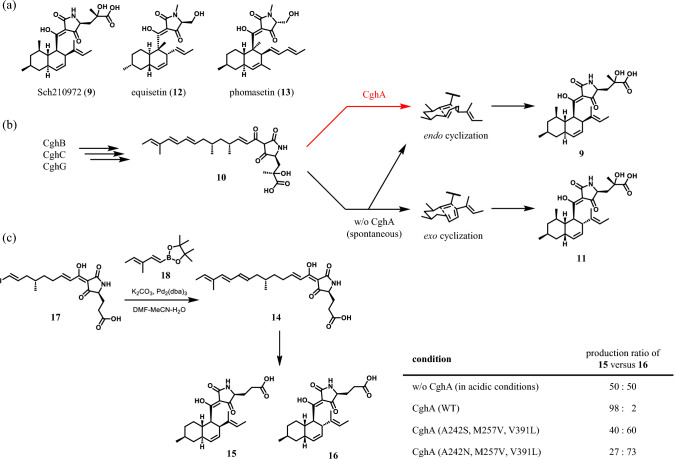
**a** Structure of Sch210972 (**9**) and related compounds. **b** Biosynthesis of **9** in the presence and absence of CghA in *Chaetomium globosum*. **c** Biochemical analysis of CghA and its mutants using simplified synthetic substrate **14**. The ratio of produced **15** versus **16** in each condition is presented in the table

Sch210972 (**9**) is an octalin-containing fungal metabolite produced by *Chaetomium globosum*. The biosynthetic pathway of **9** was elucidated by Sato and co-workers via gene deletion and heterologous expression in *Aspergillus nidulans* in 2015 [17]. CghB (aldolase), CghC (enoyl reductase), and CghG (PKS–NRPS) produced linear-chain precursor **10**, which was subsequently cyclized by CghA (Diels–Alderase) to produce **9**. Both **9** and its diastereomer **11** were detected from the *cghA*-knockout strain, suggesting **10** was non-enzymatically transformed into *endo* adduct **9** and *exo* adduct **11** (Fig. 3b). Other octalin-containing NPs such as equisetin (**12**) and phomasetin (**13**) (Fig. 3a) were also reported to be produced as a mixture of diastereomers when the corresponding Diels–Alderase genes were knocked out [18, 19]. This non-enzymatic reaction of **10** complicated biochemical characterizations of CghA: we could not obtain substrate **10** from *cghA*-knockout strain and we had to distinguish enzymatic products from non-enzymatic ones for kinetic analysis. To solve the first problem, we designed and synthesized simplified substrate **14**, which lacks two methyl groups and one hydroxyl group of **10** [20]. Spontaneous transformation of **14** to *endo*-cyclization product **15** and *exo*-cyclization product **16** was also observed during isolation, urging us to synthesize **14** just before use and use **14** without purification. Suzuki–Miyaura cross-coupling of alkenyl iodide **17** and boronic ester **18** was chosen as the last step of the synthetic scheme because this reaction creates carbon–carbon bonds in a highly regioselective manner and can be conducted in aqueous conditions (Fig. 3c).

After completion of the synthesis of **14**, point mutants of CghA were created based on the crystal structure of **9**-bound CghA, and their kinetic parameters including stereoselectivity were evaluated. In this step, the stereoselectivity of the mutants was determined at first using sufficient amounts of enzymes to prevent spontaneous reactions of **14**. Small amounts of enzymes were used for kinetic analysis to keep most of **14** unreacted, resulting in nonenzymatic production of **15** and **16** during HPLC analysis. To distinguish enzymatic products from nonenzymatic ones, we paid attention to the fact that the ratio of nonenzymatically produced **15** and **16** is 50:50. This means the variance observed between the quantities of **15** and **16** in the kinetic assay is attributed to the enzymatic products. Based on these data, the amounts of enzymatically produced **15** and **16** were calculated and kinetic parameters were determined. At the end of the work, two triple-mutants (A242S/M257V/V391L, A242N/M257V/V391L) with reversed stereoselectivity were obtained (Fig. 3c) [20]. This was the first report proving and changing the stereoselectivity of octalin-forming Diels–Alderase in the world.

## Biosynthesis of lentopeptins

**Fig. 4:**
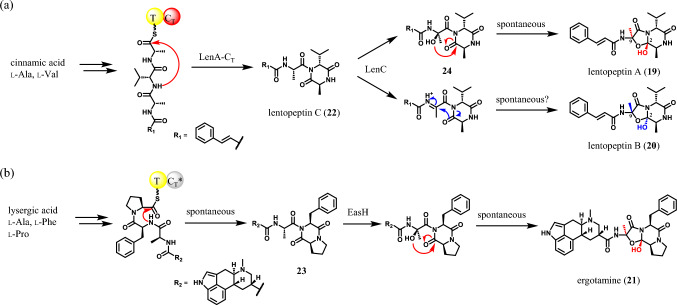
Biosynthesis of **a** lentopeptins and **b** ergotamine. *C*_T_ domain in ergotamine synthesis is inactive

Lentopeptin A (**19**) and B (**20**), produced by *Aspergillus lentulus*, possess the same planar structure but differ in stereochemistry at C-2 and C-9 (Fig. 4a) [21]. Although the structure of lentopeptins resembles that of ergotamine (**21**), **21** is produced as a single isomer in *Claviceps purpurea* (Fig. 4b) [22]. To prove what makes the difference between lentopeptins and **21**, the biosynthetic mechanism of lentopeptins was investigated. Knockout experiments revealed that the biosynthetic gene cluster (BGC) for lentopeptins is composed of only three genes: *lenA* (NRPS), *lenB* (phenylalanine-ammonia lyase) and *lenC* (P450). The biosynthesis of lentopeptins begins with the production of cinnamic acid by LenB and LenA produces mono-cyclic intermediate lentopeptin C (**22**) from cinnamic acid, L-alanine, and L-valine. Similar intermediate **23** is also produced in the biosynthesis of **21**. However, the construction of the characteristic *N*-acyl diketopiperazine moiety differs between **22** and **23**. Formation of **22** requires catalysis by terminal condensation (*C*_T_) domain of LenA at the cyclization step but **23** does not (Fig. 4) [23]. The proline residue in the linear precursor of **23** anchors the *C*-terminal thioester in proximity to the amide nitrogen, promoting spontaneous cyclization. In the last step of biosynthesis, **23** is converted only to **21** by non-heme iron dioxygenase EasH, while **22** is converted to both **19** and **20** by P450 LenC. The reaction catalyzed by LenC was examined in detail to reveal that the source of the oxygen atom incorporated during the transformation is different between **19** and **20**. In the formation of **19**, molecular oxygen is activated and added to the α-position of Ala residue in **22** to produce linear precursor **24**, which spontaneously cyclizes to form **19** in aqueous conditions. On the other hand, one of the oxygen atoms of **20** was derived from water, suggesting that **22** was dehydrogenated and hydrated to form **20**.

## Biosynthesis of fumimycin and lentofuranine

**Fig. 5:**
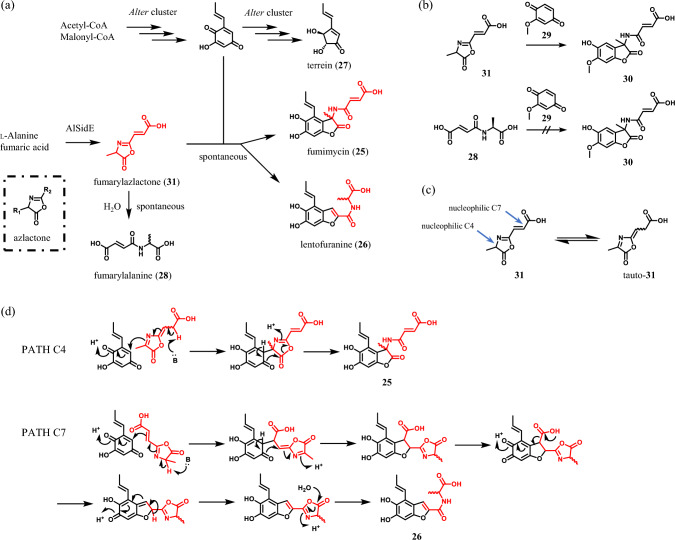
**a** Overview of the biosynthesis of fumimycin (**25**), lentofuranine (**26**) and terrein (**27**). **b** Nonenzymatic formation of fumimycin analog **30** using compound 29. **c** Unusual tautomerization of **31** to tauto-**31**. **d** Putative mechanisms of formation of **25** and **26** via nucleophilic attack from C4 (PATH C4) and C7 (PATH C7), respectively

Fumimycin (**25**), a fungal metabolite containing an unusual carbon–carbon bond between the α-carbon of alanine and an aromatic ring, was isolated from *Aspergillus fumisynnematus* in 2007 [24]. This unique structure attracted organic chemists around the world and asymmetric total synthesis of **25** was accomplished in 2010 [25]. Surprisingly, the optical rotation of natural **25** was much smaller than that of optical pure **25**, suggesting the biosynthesis of **25** involves a spontaneous racemization step. In 2023, our group isolated **25** and structurally-related compound lentofuranine (**26**) from *A. lentulus* and *Aspergillus novofumigatus* [26]. The stereochemistry of **26** was determined using Marfey’s method [27], revealing that **26** was also a racemic compound. These observations encouraged us to unveil the atypical biogenesis of **25** and **26**. However, we found no single BGC corresponding to synthesizing all the structures of **25** and **26** in the genome of *A. lentulus* and *A. novofumigatus*, indicating they are collaboratively synthesized by separated BGCs. Since **25** and **26** had the same aromatic moiety, PKS genes shared between *A. lentulus* and *A. novofumigatus* were knocked out to discover their BGC. One of the candidates was *AlterA*, which is similar to a gene corresponding to producing terrein (**27**) in *Aspergillus terreus* [28–30]. Deletion of *AlterA* in *A. lentulus* abolished the production of **25**, **26**, and **27**, revealing these three compounds share the same biosynthetic origin (Fig. 5a). Knockout analysis of the other genes in the BGC unveiled that only three genes named *AlterA* (PKS), *AlterB* (PKS), and *AlterC* (flavin-dependent monooxygenase) are indispensable for producing **25** and **26**. Judged from the function of these three, other genes located outside the BGC seemed to be necessary for the production of **25** and **26**. A gene named *AlsidE* was raised as a candidate because its ortholog *sidE* was reported to produce fumarylalanine (**28**), a compound resembling the peptidic portion of **25** and **26** [31]. As we expected, the *AlsidE*-deletion strain of *A. lentulus* could not produce **25** and **26**.

The remaining question was how AlSidE, an NRPS with A-T-C-A-T-C_T_ topology, produces these compounds. This was answered by in vitro analysis of AlSidE using compound **29** as an alternative to the quinone product of *Alter* cluster. AlSidE produced fumimycin analog **30** in addition to **28** when **29** was included in the reaction mixture. The formation of **30** was also observed in the combination of the ultrafiltrate of the AlSidE reaction mixture and **29** but not in the combination of **28** and **29** (Fig. 5b), indicating that AlSidE produced a reactive material other than **28**. We hypothesized that the reactive substance was fumarylazlactone (**31**) due to three reasons mentioned below. First, AlSidE has a C_T_ domain which is usually involved in the cyclization step of NRPS. Second, an azlactone is easily hydrolyzed to form a corresponding carboxylic acid in general. Third, an azlactone is known to racemize rapidly and **28** produced by AlSidE was racemic. To prove our hypothesis, we synthesized **31** as an authentic standard and found that AlSidE exactly produced **31**. In addition, **31** could be detected from the wild-type strain of *A. lentulus* and not from the *AlsidE*-deletion strain. These results clearly indicated that *AlsidE* is responsible for the production of **31**. To our knowledge, this was the first report of azlactone-synthesizing NRPS [26]. Since azlactones are highly reactive compounds, this naturally occurring azlactone had been overlooked for a long time. Detailed analysis of the reactivity of azlactone **31** revealed that **31** could spontaneously react with **29** to form **30** (Fig. 5b). Furthermore, **31** was found to tautomerize to an oxazolone form tauto-**31** (Fig. 5c), indicating both C-4 and C-7 are nucleophilic. Based on these chemical properties of **31**, we proposed the mechanisms of formation of **25** and **26** as shown in Fig. 5d. Interestingly, **31** was also produced by other *Aspergillus* and *Penicillium* fungi lacking an *AlterA* ortholog necessary for producing **25** and **26**, suggesting that **31** itself would play some roles in the lifecycle of the producer as discussed in the discovery of natural oxazolones by Rond et al. [32].

## Conclusion

This mini review has highlighted four types of NP biosynthesis accompanying non-enzymatic reactions. In the case of viridicatins and Sch210972, non-enzymatic reactions caused problems in the analysis of enzymatic reactions. However, these problems could be solved by changing reaction conditions to avoid non-enzymatic ones. Hence understanding what drives spontaneous reactions and how they can be prevented are important in biosynthetic study. On the other hand, non-enzymatic reactions are essential in the biosynthesis of lentopeptins and lentofuranine. In these cases, analysis of the reactivity of enzymatically produced intermediates was the key to uncovering the true mechanism of biosynthesis. Due to the large number of NP biosyntheses remaining to be elucidated, there could be numerous undiscovered non-enzymatic reactions in nature. It is important for researchers to carefully assess what is going on during the biosynthesis.
